# Supplementary material for: A Novel in Duck Myoblasts: The Transcription Factor Retinoid X Receptor Alpha (RXRA) Inhibits Lipid Accumulation by Promoting CD36 Expression
Source: Int J Mol Sci. 2023 Jan 7;24(2):1180. doi: 10.3390/ijms24021180 (PMC9864336; doi:10.3390/ijms24021180)
Supplement: Supplementary file 1 [file ijms-24-01180-s001.zip › Table S1.pdf]

Table S1. List of the primer sequences used for RT–PCR.

Genes		Primer sequences (5′-3′)	Product/bp	Tm/℃
RXRA	F	TGCGGAAGGACCTGACCTACAC	178	60
	R	GACTCCACCTCGTTCTCGTTGC		
CD36	F	GCTTGTGCCAGGAAGCTCAGTT	268	60
	R	AGCAACAACAGCGAGGTTCAGG		
ACSL1	F	CGTTACTCCACCGAGGCTTCAA	115	60
	R	TCATAGAGCGGCACCACTACCA		
ELOVL6	F	CAGTCAGTGTGCGACCAGAGTT	226	60
	R	CCAGCCACCATGTCCTTGTAGG		
FABPA	F	GCTGGGTGTGGGATTTGCTA	168	60
	R	CTGTCATCTGCTGTGGTCTCA		
PPARG	F	GCCACAAGCGGAGAAGGAGAAG	186	60
	R	GCAGCGGTGACACATGCTTACA		
GAPDH	F	GGAGAAACCAGCCAAGTAT	177	60
	R	CCATTGAAGTCACAGGAGA		
