# Supplementary material for: A Novel in Duck Myoblasts: The Transcription Factor Retinoid X Receptor Alpha (RXRA) Inhibits Lipid Accumulation by Promoting CD36 Expression
Source: Int J Mol Sci. 2023 Jan 7;24(2):1180. doi: 10.3390/ijms24021180 (PMC9864336; doi:10.3390/ijms24021180)
Supplement: Supplementary file 1 [file ijms-24-01180-s001.zip › Table S2.pdf]

Table S2. Sequences of the primers for gene coding region amplification.

Genes		Primer sequences (5′ - 3′)	Restriction site	Product/bp	Tm/℃
RXRA	F	GCTAGCATGGACACCAAACACTTCCTG	NheI (GCTAGC)	1416	68
	R	AAGCTTCTAAGTCATTTGATGGGGCG	HindIII (AAGCTT)		
CD36	F	GGATCCATGGGCTGTAACCGAAACT	BamHI (GGATCC)	1428	62.5
	R	GCTAGCTTATTTTAATTTCTTTGATCTGC	NheI (GCTAGC)		
