# Supplementary material for: A Novel in Duck Myoblasts: The Transcription Factor Retinoid X Receptor Alpha (RXRA) Inhibits Lipid Accumulation by Promoting CD36 Expression
Source: Int J Mol Sci. 2023 Jan 7;24(2):1180. doi: 10.3390/ijms24021180 (PMC9864336; doi:10.3390/ijms24021180)
Supplement: Supplementary file 1 [file ijms-24-01180-s001.zip › Table S3.pdf]

Table S3. List of shRNA sequences.

Name	Sequence (5′-3′)
RXRA-shRNA1	CCGGGGACAGGTCTTCAGGTAAACACTCGAGTGTTTACCTGAAGACCTGTCCTTTTTG
RXRA-shRNA2	CCGGGCGTACTGCAAGCACAAATACCTCGAGGTATTTGTGCTTGCAGTACGCTTTTTG
RXRA-shRNA3	CCGGGCTCCCACATTGCTGTAAATCCTCGAGGATTTACAGCAATGTGGGAGCTTTTTG
RXRA-shRNA4	CCGGGGATTTCAGGTCGTAACTTTGCTCGAGCAAAGTTACGACCTGAAATCCTTTTTG
CD36-shRNA1	CCGGGGACAGGTCTTCAGGTAAACACTCGAGTGTTTACCTGAAGACCTGTCCTTTTTG
CD36-shRNA2	CCGGGCGTACTGCAAGCACAAATACCTCGAGGTATTTGTGCTTGCAGTACGCTTTTTG
CD36-shRNA3	CCGGGCTCCCACATTGCTGTAAATCCTCGAGGATTTACAGCAATGTGGGAGCTTTTTG
CD36-shRNA4	CCGGGGATTTCAGGTCGTAACTTTGCTCGAGCAAAGTTACGACCTGAAATCCTTTTTG
