# Supplementary figures and images for: A Novel in Duck Myoblasts: The Transcription Factor Retinoid X Receptor Alpha (RXRA) Inhibits Lipid Accumulation by Promoting CD36 Expression
Source: Int J Mol Sci. 2023 Jan 7;24(2):1180. doi: 10.3390/ijms24021180 (PMC9864336; doi:10.3390/ijms24021180)

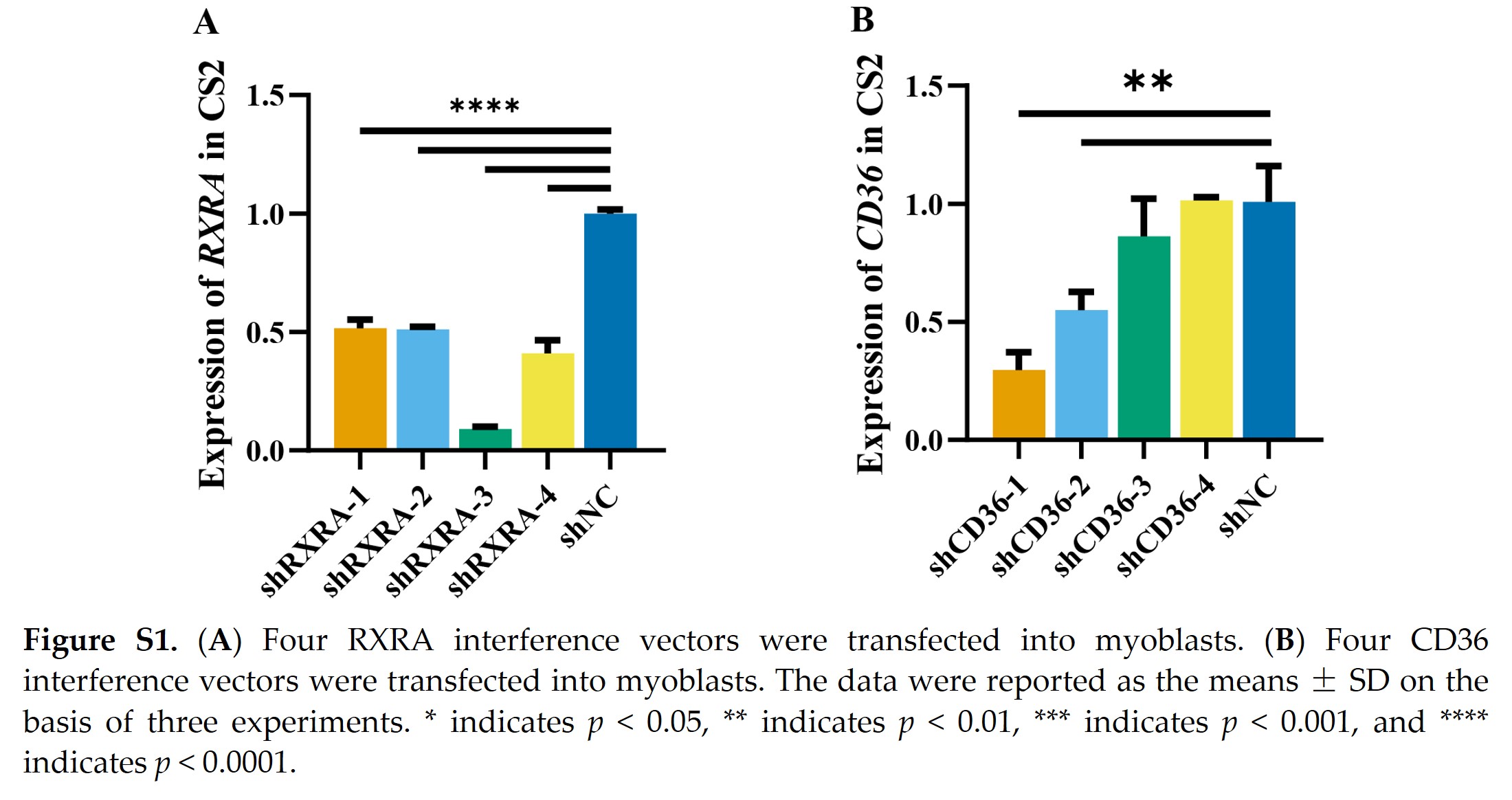

Supplement: Supplementary file 1 [file ijms-24-01180-s001.zip › Figure S1.jpg]

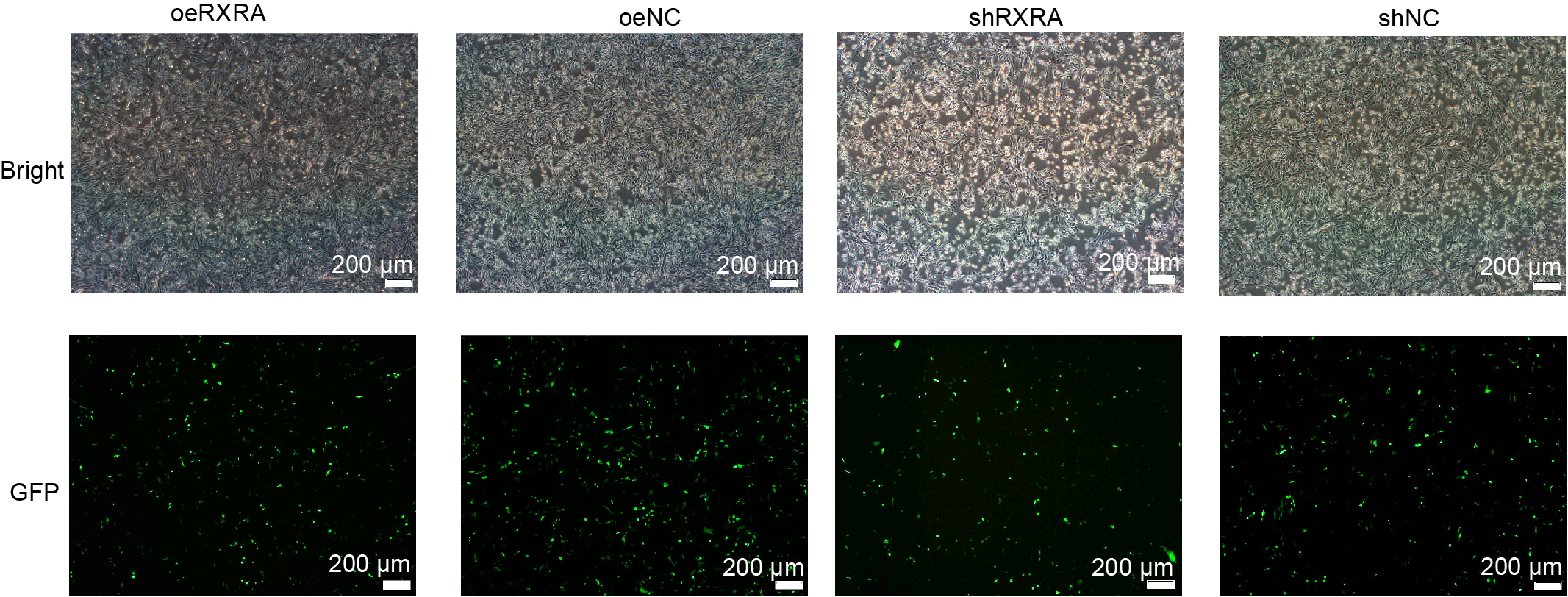

Supplement: Supplementary file 1 [file ijms-24-01180-s001.zip › Figure S2.jpg]

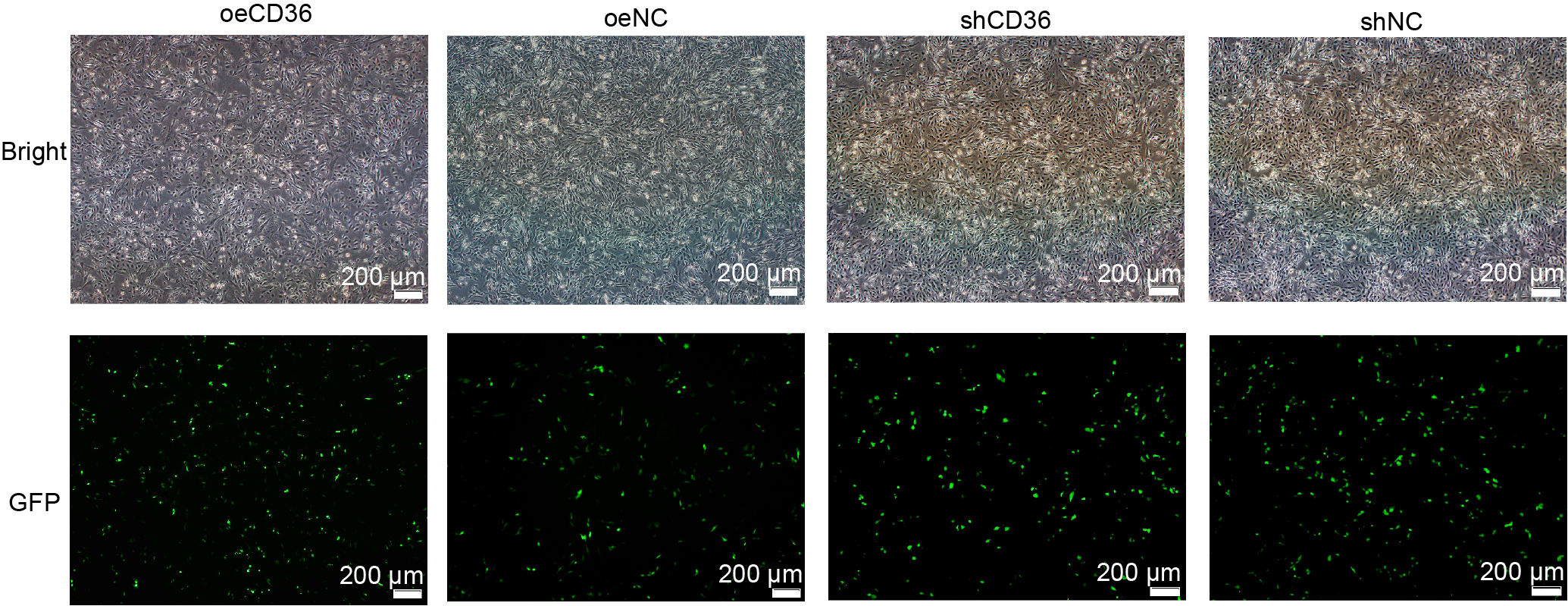

Supplement: Supplementary file 1 [file ijms-24-01180-s001.zip › Figure S3.jpg]

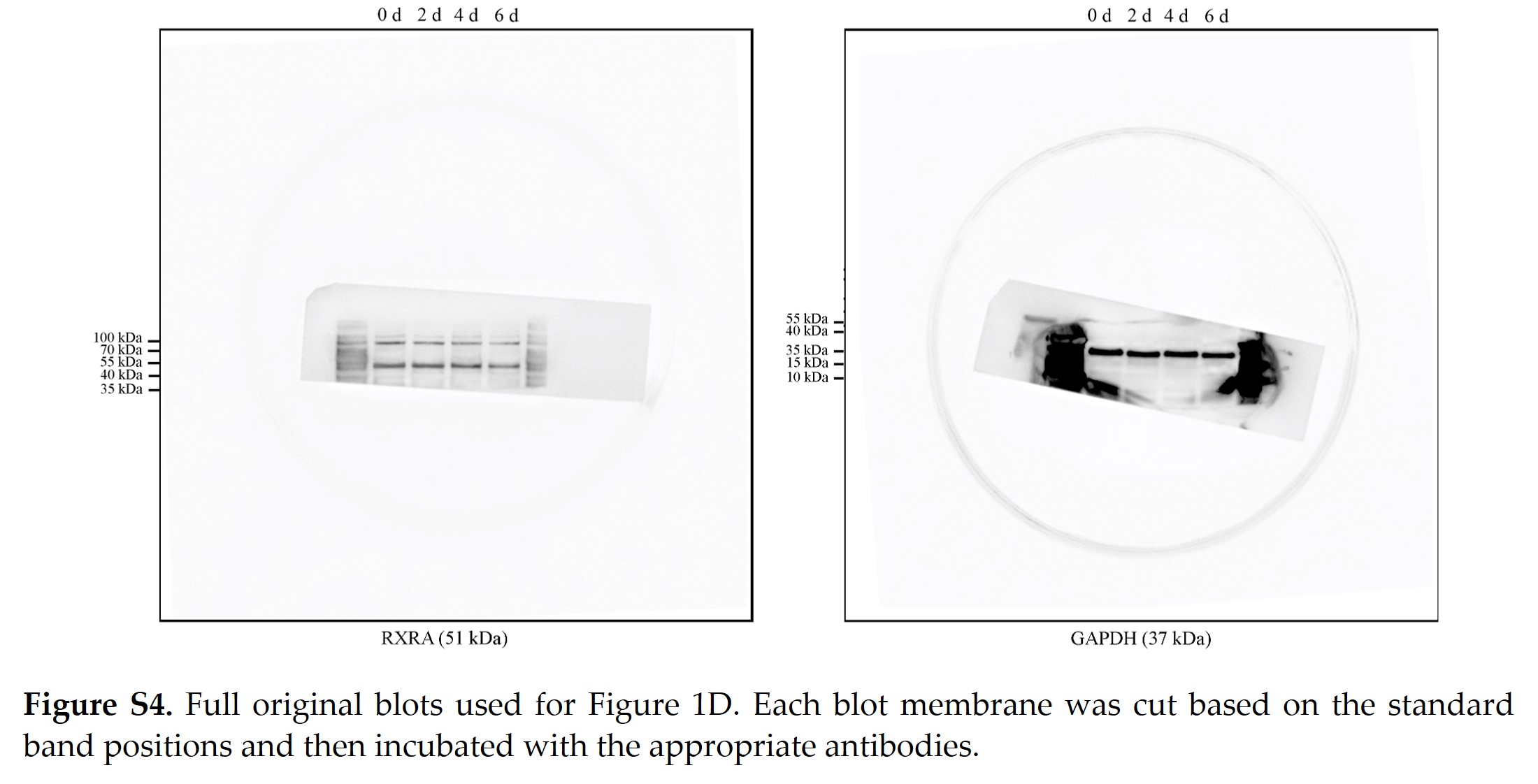

Supplement: Supplementary file 1 [file ijms-24-01180-s001.zip › Figure S4.jpg]

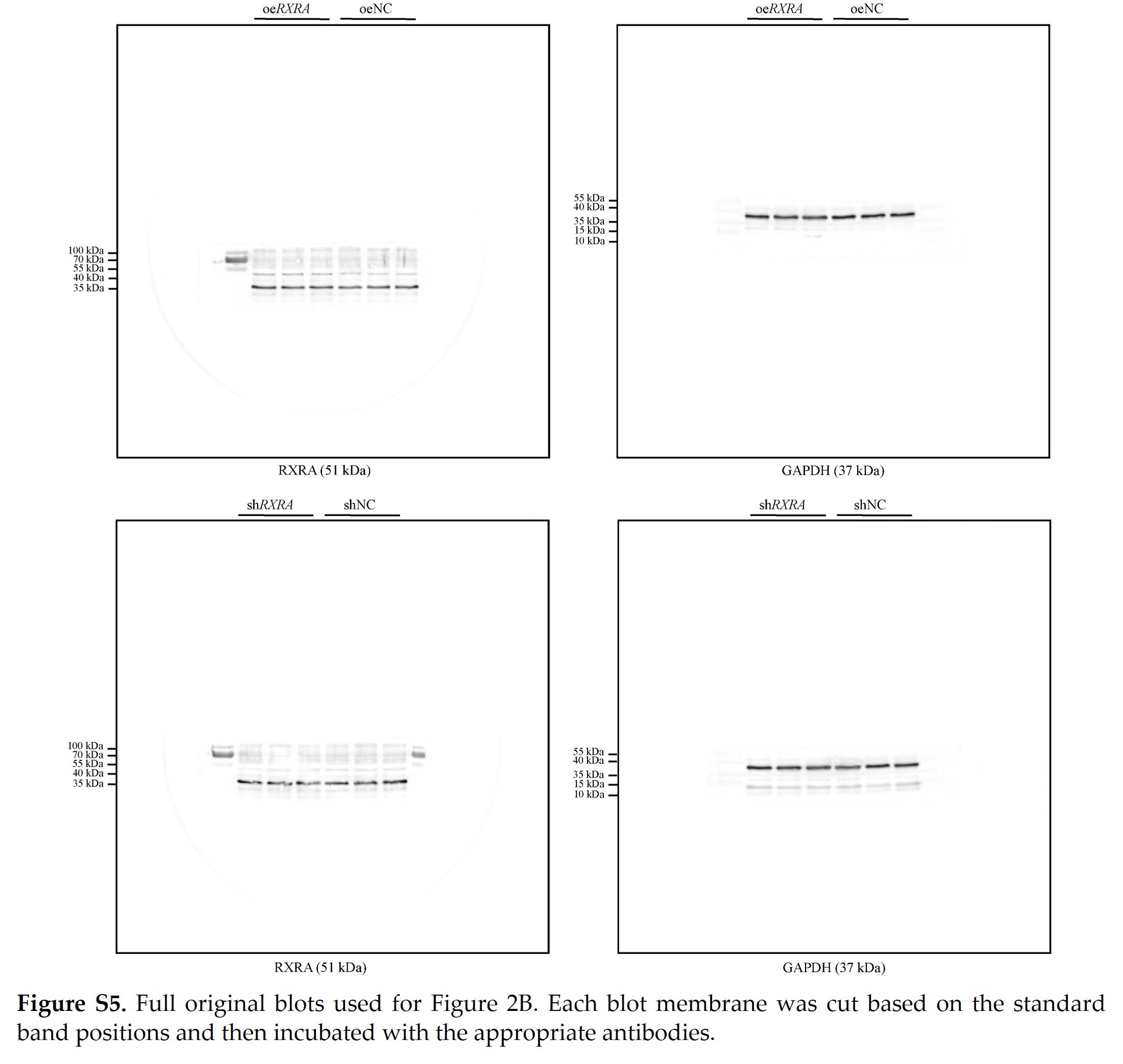

Supplement: Supplementary file 1 [file ijms-24-01180-s001.zip › Figure S5.jpg]

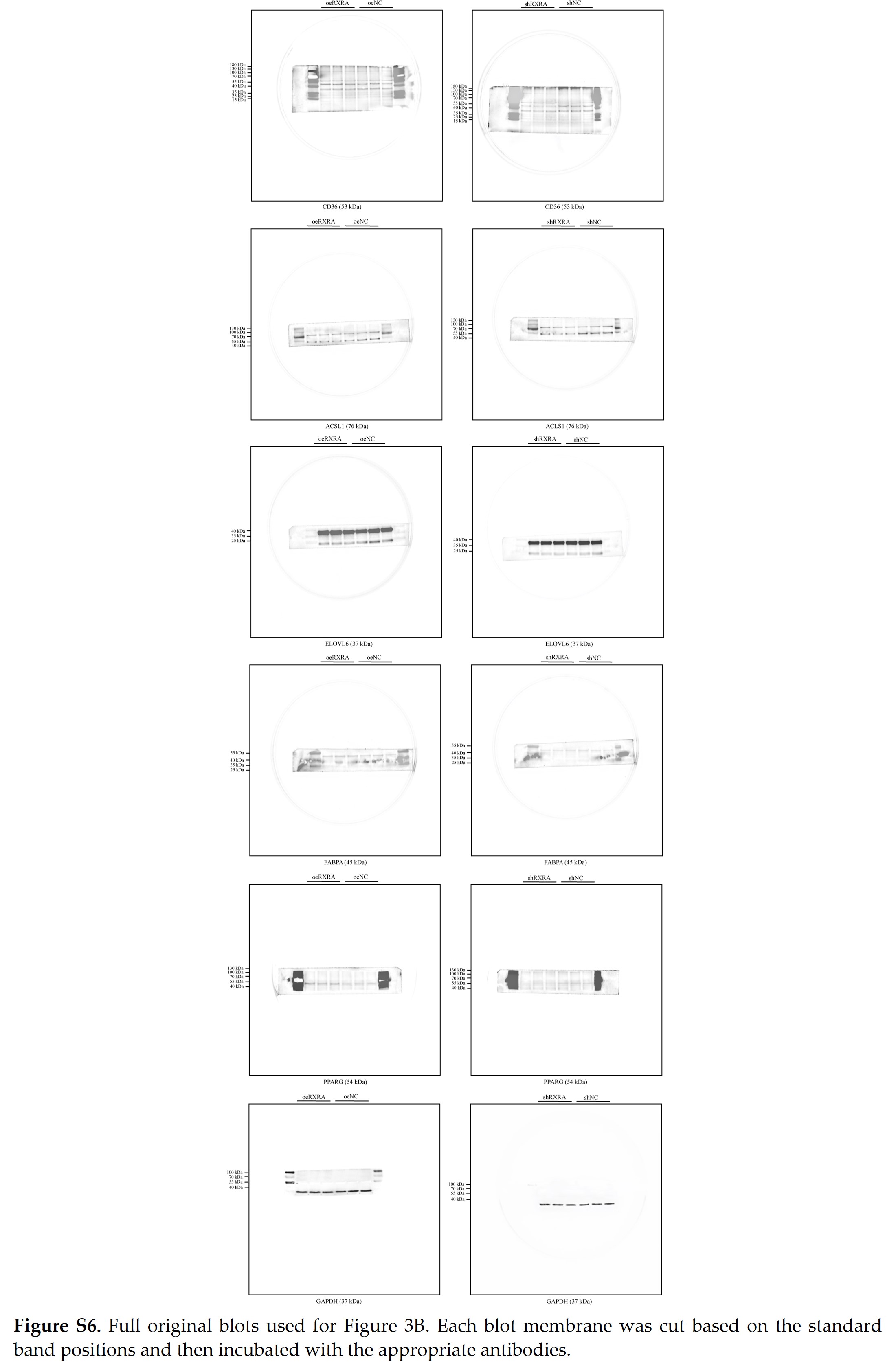

Supplement: Supplementary file 1 [file ijms-24-01180-s001.zip › Figure S6.jpg]

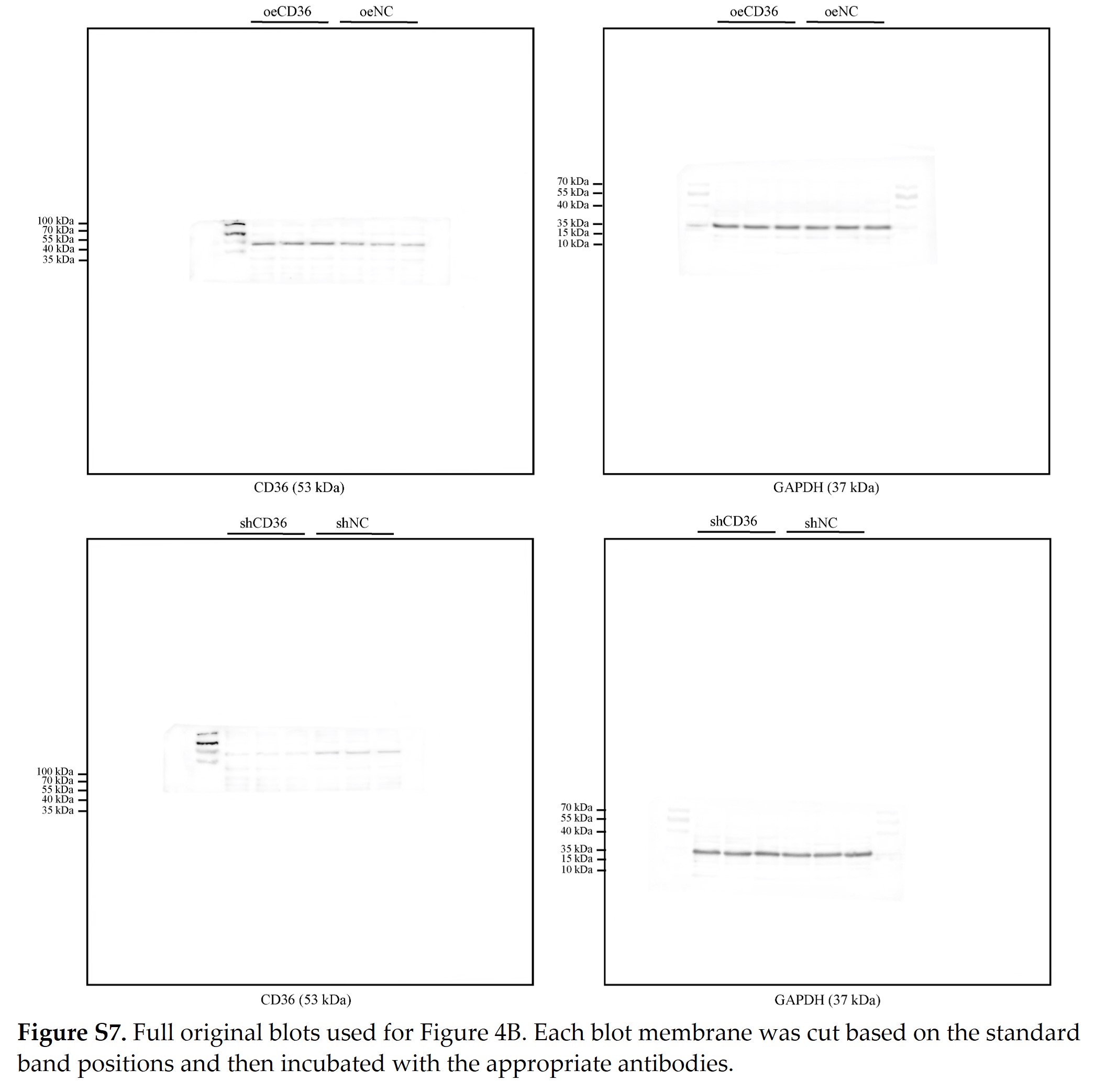

Supplement: Supplementary file 1 [file ijms-24-01180-s001.zip › Figure S7.jpg]
